# Associations between the use of insecticide-treated nets in early childhood and educational outcomes, marriage and child-bearing in early adulthood: evidence from a 22-year prospective cohort study in Tanzania

**DOI:** 10.1186/s12936-023-04560-z

**Published:** 2023-04-25

**Authors:** Sigilbert Mrema, Fredros Okumu, Joanna Schellenberg, Günther Fink

**Affiliations:** 1 Ifakara Health Institute, Ifakara, Tanzania; 2 London School of Hygiene and Tropical Medicine, London, UK; 3 Swiss Tropical and Public Health Institute and University of Basel, Kreuzstrasse 2, Allschwil, 4123 Basel, Switzerland

**Keywords:** Early life, Insecticide treated net, Early adulthood development, Educational attainment, Marriage, Childbearing

## Abstract

**Background:**

The effectiveness of insecticide-treated nets (ITNs) in preventing malaria in young children is well established. However, the long-term effects of early childhood ITN use on educational outcomes, fertility, and marriage in early adulthood are not well understood.

**Methods:**

This study uses 22 years of longitudinal data from rural Tanzania to investigate the associations between early life ITN use and educational attainment, fertility and marriage in early adulthood. Unadjusted and adjusted logistic regression models were used to estimate the associations between early life ITN use and early adult outcomes (education, childbearing, and marriage), controlling for potential confounders, such as parental education, household asset quintiles, and year of birth. Analyses were conducted separately for men and women.

**Results:**

A total of 6706 participants born between 1998 and 2000 were enrolled in the study between 1998 and 2003. By 2019 a total of 604 had died and a further 723 could not be found, leaving 5379 participants who were interviewed, among whom complete data were available for 5216. Among women, sleeping under a treated net at least half of the time during early childhood [“high ITN use”] was associated with a 13% increase in the odds of completing primary school (adjusted odds ratio (aOR) 1.13 [0.85, 1.50]) and with a 40% increase in the odds of completing secondary school (aOR 1.40 [1.11, 1.76]) compared with women sleeping less frequently under ITNs in early life (< age 5 years). Among men, high ITN use was associated with a 50% increase in the odds of completing primary school (aOR 1.50 [1.18, 1.92]) and a 56% increase in the odds of completing secondary school (aOR 1.56 [1.16, 2.08]) compared to men with low ITN use in early life. Weaker associations were found between ITN use in early life and both adolescent childbearing (aOR 0.91 [0.75, 1.10]) and early marriage (aOR 0.86 [0.69, 1.05]).

**Conclusion:**

This study found that early life use of ITNs was strongly associated with increased school completion in both men and women. More marginal associations were found between early-life ITN use and both marriage and child-bearing in early adulthood. ITN use during early childhood may have long-term positive effects on educational attainment in Tanzania. However, further research is needed to understand the mechanisms behind these associations and to explore the broader impacts of ITN use on other aspects of early adult life.

**Supplementary Information:**

The online version contains supplementary material available at 10.1186/s12936-023-04560-z.

## Background

Malaria is considered a threat to recent gains in health and development and to the attainment of the 2030 agenda for Sustainable Development [1]. There were 620,000 malaria deaths in 2021, more than 90% of this burden being in sub-Saharan Africa (sSA), and more than 80% among children below 5 years of age [2]. Like other countries in sSA, Tanzania was involved in the research process establishing insecticide-treated nets (ITNs) as a malaria control tool since the 1980s [3]. In early 2000s, ITNs were declared as a primary tool for preventing the disease in Africa [4]. Since then, Tanzania has made major efforts to expand the access to and use of ITNs. Reports indicate that the proportion of households in Tanzania owning at least one ITN has increased from 14% to 2001 to 74% in 2022, even though ITN use remains lower in rural than in urban areas [5]. More than 2 billion ITNs have been distributed through public or private sector channels globally [2] and likely explain most of the gains accrued against malaria since 2000 [6, 7].

There are both short- and long-term benefits of using ITNs [2, 8]. A recent study from Tanzania, linked to the current paper, reported that consistent use of ITNs in early childhood brings a survival advantage not only in childhood but also up to adulthood [9]. A growing body of literature has analysed the relationships between early life exposure to malaria and adult human capital outcomes. Malaria infections in children have been linked to reduced cognitive and socio-emotional development in childhood [10, 11], as well as poor school performance [12] and reduced educational attainment [13]. Reduced educational attainment has been linked to an increased risk of early marriage [14, 15] and teenage pregnancies [16]. According to the latest data, 46% of 19 year old women in Tanzania report to have ever been pregnant, and only 30% of women ages 15–49 attained secondary schooling or higher education [5]. Early life exposure to malaria has also been linked to reduced adult incomes [17] as well as an increased risk of cardiovascular disease and poorer cognitive performance in old age [18]. It however remains unclear to what extent ITN use in childhood can reduce the risk of such adverse outcomes in later life.

This paper reports results from a 22 year prospective cohort study in rural south-eastern Tanzania, to estimate the associations between the use of ITNs in early childhood and socio-demographic development indicators, including educational attainment, marital status and having children, in young adult men and women.

## Methods

### Study design

This was a prospective cohort study, following participants from early childhood (< 5 years of age) to early adulthood (18–21 years old).

### Setting

**Fig. 1:**
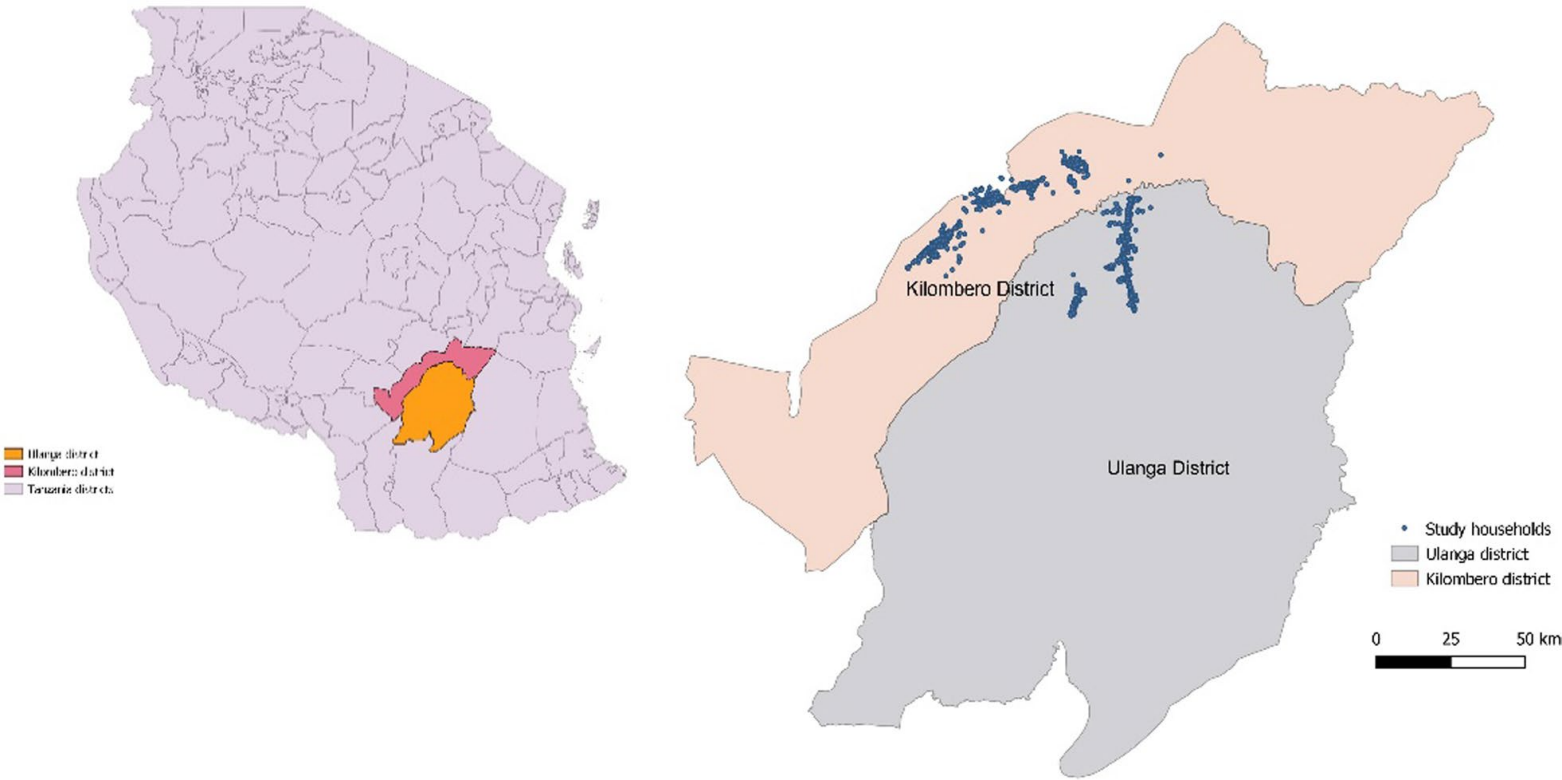
The left hand figure shows the location of Ulanga and Kilombero within Tanzania. The right hand figure shows the location of study households within these two districts

The cohort of this study was originally set up as part of the Kilombero Net Programme (KINET). The KINET programme was nested within the Ifakara rural Health and Demographic Surveillance Site (HDSS) between 1997 and 2000 to explore community-wide benefits of ITNs on both morbidity and mortality [3]. The HDSS is located in the Kilombero and Ulanga districts in Tanzania (Latitude: 8^0^00’S–8^0^35 S; Longitude: 35^0^58’E–36^0^48’E and Elevation: 270–1000 m). The study area (Fig. 1) comprised of 25 villages, and a geographical area of 2400 square kilometers to the West and South of Ifakara town [19]. Additional details of the study setting are reported elsewhere [9].

### Recruitment, enrolment, follow-up, and sample size

From its inception in 1997 to 2013 the Ifakara HDSS included three rounds of household visits each year, changing to two rounds per year from 2014 to 2016. During the household visits conducted between May 1998 and April 2003, all children born between January 1st, 1998 and August 30th, 2000 to an HDSS resident were recruited to the study and monitored through the HDSS visits every 4 months. From 2004 to 2015, for children remaining in the area, survival status and socio-demographic development were again monitored through the HDSS system. At enrolment, interviewers asked the mothers or caregivers to provide information such as date and place of birth, as well as the sex of the child. At HDSS household visits up to 2003, parents or care givers were asked whether the child had slept under a net the previous night and about net treatment and washing as well as about a range of potential confounders, including parental education and household assets, distance to health facility and water and sanitation access.

In 2019, a follow-up survey was conducted to verify the survival status and socio demographic development of all participants, regardless of where they were living at that time, thus including those who had out-migrated during the study period. The 2019 follow-up survey collected information about members of the original households, including their names and ages, as well as the name of their local leader (*Balozi*) listed at the time of enrolment. Separate search lists were created for each of the 25 villages in the HDSS, and meetings were held with village leaders to review these lists and to identify community members who could help with additional tracking. Additional information collected during this follow-up survey included educational attainment of individual participants, their marital status, and whether they had already had children.

### Variables

The primary outcome variables of interest were: educational attainment, marital status and childbearing at the time of the 2019 follow-up survey. For educational attainment, the analysis included: whether the participant was still in school when interviewed in 2019, whether they had completed at least 7 years of education (primary school or higher), and whether they had completed 11 or more years of education (secondary school or higher). For childbearing and marriage, the analysis used binary indicators for respondents reporting to be married or having had a child at the time of the interview. Given that respondents were between 18 and 21 years old at the time of the follow-up, these outcome measures do not correspond exactly to adolescent fertility or teenage marriage but serve to approximate early transitions into marriage and parenthood.

The primary independent variable of interest was early life use of ITNs, estimated from individual use of nets reported during up to 15 household visits between 1998 and 2003. At each visit, the caregivers were asked whether the child had slept under a net the previous night before each visit, and, if the answer was positive, whether the net used was an untreated or treated net. The primary exposure variable used was a binary variable categorizing each child as having slept under an ITN at under 50% of early-life visits or at 50% or more of early-life visits.

In the sensitivity analysis, two alternative measures of early life treated net use were used: first, the (continuous) percentage of visits for which the caregivers reported the child having slept under a treated net (ranging between 0 and 100); second, a restricted sample of children who were either always or never reported to have slept under a treated net was analysed. Additional file 1: Fig. S1 illustrates the empirical distribution of early life treated net usage, showing that children using nets 50% of the time or more constitute roughly half of the sample.

To address potential confounders, additional information on parent or caregiver education, household asset quintiles and participant’s year of birth collected during the 1998 to 2003 visits were included in the empirical models analysed.

### Managing biases

The primary concern with the follow-up were biases resulting from attrition given the long follow-up period. To minimize such concerns, an experienced team of field workers was hired, who were closely familiar with the study villages. To ensure team motivation, financial bonuses were offered if 60, 70 and 80% of young adults could be found overall.

Given the focus on ITNs, the main bias concern for the analysis was confounding. Even though the rapid scaleup in the region was mostly driven by external forces, it seems likely that initial net ownership was higher among more highly educated and wealthier families whose children generally experience better educational outcomes. To address these concerns, adjusted models were used, controlling for parental education and household assets.

### Statistical methods

First, basic descriptive statistics were generated, describing ITN use by year of birth, sex, caregiver education, marital status, having children and household income categories. Educational attainment was then presented, by gender, as the primary outcome. Univariate and multivariate logistic regression analyses were done to assess the associations between early life use of ITNs and early adulthood outcomes. All statistical analysis was conducted in Stata SE 16.0; 95% confidence intervals were used to show uncertainty in the estimates.

## Results

**Fig. 2:**
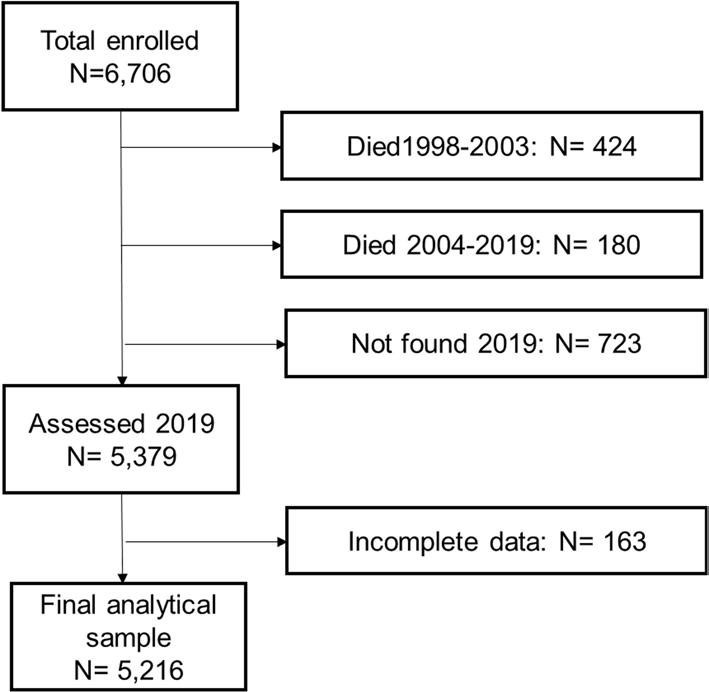
Recruitment, enrolment and follow-up

Figure 2 presents the flow chart of enrolment of study participants together with numbers who were reached during the follow-up study in 2019. Of the 6706 children enrolled in the study between 1998 and 2003, 5379 were found again in 2019; and complete data was obtained for 5216 participants.

**Fig. 3:**
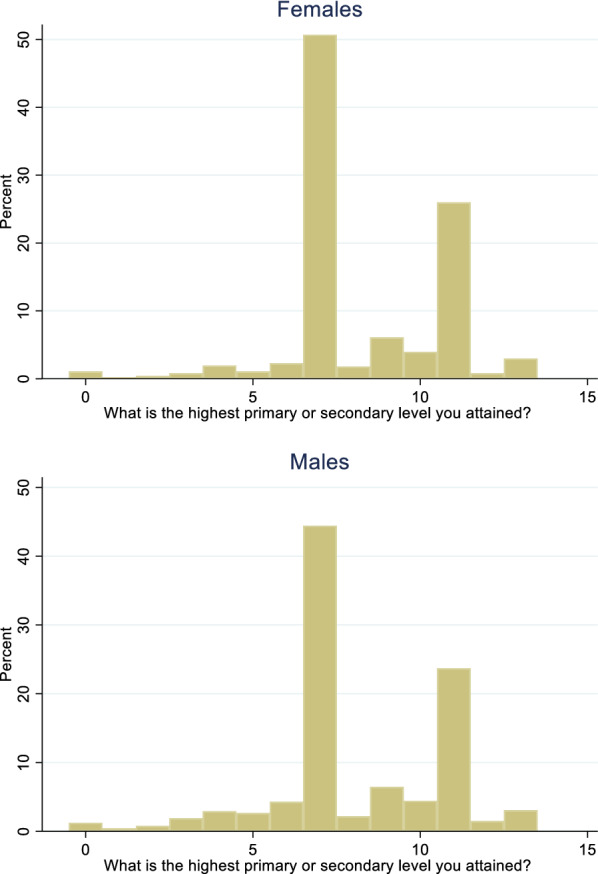
Highest level of education attained for males and females

Table 1 presents the characteristics of these 5216 study participants, grouped according to their early-life use of ITNs. In the 2019 follow-up survey, 57% of study participants were living with their parents, and 16% were still in school. Most study participants (89%) had completed at least 7 years of primary education; 29% had completed 11 years, i.e. primary school and 4 years of secondary school. The distribution of educational attainment is shown in Fig. 3, with peaks at 7 and 11 years corresponding to completion of primary and the first 4 years of secondary education, respectively. Overall, 21% of participants were married and 24% had children. The rate of marriage and childbearing in this cohort of 18–21 year-olds was 5 times higher among women than men.

**Table 1:** Descriptive characteristic of the study participants by treated net coverage

Characteristics	Treated net use < 50%				Treated net use ≥ 50%					
	Females		Males		Females		Males		Overall	
Year of birth	N	%	N	%	N	%	N	%	N	%
Cohort 1998	414	42	369	41	622	38	610	37	2015	39
Cohort 1999	356	36	331	37	620	37	583	35	1890	36
Cohort 2000	211	22	206	22	422	25	472	28	1314	25
Living status										
Lives with parents	430	44	599	66	820	49	1112	67	2961	57
Educational attainment										
< 7 years completed	85	9	158	17	119	7	207	13	569	11
7 − 10 years completed	644	66	541	60	1008	61	936	56	3132	60
11 years plus completed	252	25	207	23	537	32	522	31	1518	29
Ongoing education										
Still studying	118	12	121	13	236	14	348	21	823	16
Marriage and children										
Married	364	37	76	8	537	32	101	6	1081	21
Has children	442	45	61	7	661	40	106	6	1273	24
Caretaker education										
None	160	16	108	12	162	10	157	9	587	11
Some primary	242	25	235	26	359	22	338	20	1175	23
Primary completed	533	54	523	58	1058	64	1080	65	3195	61
Secondary or more	24	3	22	2	58	4	48	3	152	3
Wealth quintile										
Quintile 1 (poorest)	270	29	248	29	271	17	269	16	1059	21
Quintile 2	161	17	159	18	257	16	243	15	821	16
Quintile 3	203	21	188	22	426	26	413	25	1230	24
Quintile 4	146	15	134	15	242	15	241	15	763	15
Quintile 5 (least poor)	167	18	139	16	424	26	472	29	1203	24

On average, women who had slept under treated nets at the majority of early-life visits had 23% higher odds of attaining 7 or more years of education [OR: 1.23; 95% CI 1.02, 1.48] and 38% higher odds of attaining 11 or more years of education [OR: 1.38; 95% CI 1.08, 1.74], compared to those who slept under the nets for less than half of the early-life visits (Table 2). Women who had slept under treated nets at the majority of early-life visits were 19% less likely to be married [OR: 0.81; 95% CI 0.67, 0.97] and 20% less likely to have children [OR: 0.80; 95% CI 0.65, 0.98] compared to those with lower early-life use of treated nets. Likewise, men who had slept under ITNs at the majority of early-life visits had 71% higher odds of still being in school [OR: 1.71; 95% CI  1.29, 2.26], 49% higher odds of attaining 7 or more years of education [OR: 1.49; 95% CI 1.20, 1.84] and 54% higher odds of attaining 11 or more years of education [OR: 1.54; 95% CI 1.23, 1.93], respectively as compared to those with lower early-life use of treated nets. In addition, men who had slept under treated nets at the majority of early-life visits were 29% less likely to be married [OR: 0.71; 95% CI 0.51, 0.97] and 6% less likely to have children [OR: 0.94; 95% CI 0.68, 1.29] as compared to those with lower early-life use of treated nets.

**Table 2:** Early life use of treated nets and sociodemographic outcomes in adulthood

Variables	(1)	(2)	(3)	(4)	(5)
	In school	7 years plus	11 years plus	Married	Has children
Female sample					
Treated net use reported during <50% of visits (ref)	1.00	1.00	1.00	1.00	1.00
Treated net reported during ≥50% of visits	1.21	1.23	1.38	0.81	0.80
	(0.86, 1.69)	(1.02, 1.48)	(1.08, 1.74)	(0.67, 0.97)	(0.65, 0.98)
Observations	2645	2645	2645	2645	2645
Males sample					
Treated net use reported during <50% of visits (ref)	1.00	1.00	1.00	1.00	1.00
Treated net use reported during ≥50% of visits	1.71	1.49	1.54	0.71	0.94
	(1.29, 2.26)	(1.20, 1.84)	(1.23, 1.93)	(0.51, 0.97)	(0.68, 1.29)
Observations	2571	2571	2571	2571	2571
Combined sample					
Treated net use reported during <50% of visits (ref)	1.00	1.00	1.00	1.00	1.00
Treated net use reported during ≥50% of visits	1.47	1.36	1.45	0.78	0.82
	(1.13, 1.90)	(1.22, 1.51)	(1.21, 1.72)	(0.67, 0.90)	(0.70, 0.96)
Observations	5216	5216	5216	5216	5216

Table 2 shows unadjusted logistic regression results estimating the associations between early life treated net use and sociodemographic outcomes in adulthood. Panel A shows results for women, panel B for men and panel C for both women and men. All presented estimates are odds ratios from univariate logistic regression with 95% confidence intervals in parentheses. No control variables are included in these unadjusted models

Combining men and women (panel C) shows that young adults who slept under treated nets at the majority of early-life visits were 47% more likely to still be in school [OR: 1.47 95% CI 1.13, 1.90], 36% more likely to have attained 7 or more years of education [OR: 1.36; 95% CI 1.22, 1.51] and 45% [OR: 1.45; 95% CI 1.21, 1.72] more likely to have attained 11 or more years of education compared to those with lower early-life use of treated nets. Similarly, young adults who slept under treated nets at the majority of their early-life visits had 22% reduced odds of being married [OR: 0.78; 95% CI 0.67, 0.90] and 18% lower odds of having children [OR: 0.82; 95% CI 0.70, 0.96] compared to those with lower early-life use of treated nets.

Table 3 shows the results of five separate adjusted regression models (in school, 7 or more years education, 11 or more years education, married and has children) estimating the association between early life use of ITNs and each of these five outcomes, controlling for wealth quintile, parents education and year of birth. For women, consistent use of ITNs in early-childhood was associated with 40% higher odds of attaining 11 or more years of education [adjusted odds ratio (aOR): 1.40; 95% CI 1.11, 1.76]. Early-life use of treated nets was less strongly associated with being in school [aOR: 1.15; 95% CI 0.76, 1.76], and with having attained 7 or more years of education [AOR: 1.13; 95% CI 0.85, 1.50]. Similarly for marriage and having children, in adjusted models, women had 9% decreased odds of being married [aOR: 0.91; 95% CI 0.69, 1.19] and 11% decreased odds of having children [aOR: 0.89; 95% CI 0.72, 1.10] comparing higher early-life net use to lower early-life use.

**Table 3:** Early life use of treated nets and sociodemographic outcomes in adulthood

Variables	(1)	(2)	(3)	(4)	(5)
	In school	7 years plus	11 years plus	Married	Has children
Panel A. Females					
Treated net use reported during <50% of visits (ref)	1.00	1.00	1.00	1.00	1.00
Treated net use reported during ≥50% of visits	1.15	1.13	1.40	0.91	0.89
	(0.76, 1.76)	(0.85, 1.50)	(1.11, 1.76)	(0.69, 1.19)	(0.72, 1.10)
Observations	2552	2494	2567	2567	2564
Panel B. Males					
Treated net use reported during <50% of visits (ref)	1.00	1.00	1.00	1.00	1.00
Treated net use reported during ≥50% of visits	1.52	1.50	1.56	0.72	0.98
	(1.10, 2.09)	(1.18, 1.92)	(1.16, 2.08)	(0.45, 1.14)	(0.66, 1.46)
Observations	2456	2497	2498	2424	2417
Panel C. Both sex					
Treated net use reported during <50% of visits (ref)	1.00	1.00	1.00	1.00	1.00
Treated net use reported during ≥50% of visits	1.342	1.347	1.474	0.858	0.910
	(1.00, 1.79)	(1.14, 1.59)	(1.23, 1.76)	(0.69, 1.05)	(0.75, 1.10)
Observations	5036	5055	5073	5073	5069

Table 3 shows adjusted logistic regression results estimating the associations between early life treated net use and sociodemographic outcomes in adulthood. Panel A shows results for women, panel B for men and panel C for both women and men. All presented estimates are odds ratios from univariate logistic regression with 95% confidence intervals in parentheses. All models include controls for wealth quintile (indicators for the 2nd, 3rd, 4th and 5th quintile), caregiver education (some primary, primary completed, secondary of higher) and year of birth (1999, 2000)

For men, after controlling for wealth quintile, caregiver education and year of birth, early-life use of treated nets was associated with 52% increased odds of being in school [aOR: 1.52; 95% CI 1.10, 2.09], 50% increased odds of attaining 7 or more years of education [aOR: 1.50; 95% CI 1.18, 1.92] and 56% increased odds of attaining 11 or more years of education [aOR: 1.56; 95% CI 1.16, 2.08] as compared to those with lower early-life coverage. In adjusted models, among men, higher early-life net use was associated with 28% decreased odds of being married [aOR: 0.72; 95% CI 0.45, 1.14] and 2% decreased odds of having children [aOR: 0.98; 95% CI 0.66, 1.46] compared to lower early-life use. Adjusted models yielded more moderate effect sizes for demographic outcomes than unadjusted models, suggesting some confounding for these measures.

For both men and women combined, after controlling for wealth quintile, caregiver education and year of birth, subjects who had slept under treated net at the majority of early-life visits had 34% higher odds of still schooling [aOR: 1.34; 95% CI 1.00, 1.79] than the lower use group. Higher use was also associated with 34% higher odds of attaining 7 or more years of education [aOR: 1.34; 95% CI 1.14, 1.59], and with 47% higher odds of attaining 11 or more years of education [aOR: 1.47; 95% CI 1.23, 1.76]. Less marked evidence of associations were found between early life bed net use and marriage [aOR: 0.85; 95% CI 0.69,1.05] or childbearing [aOR: 0.91; 95% CI 0.75, 1.10].

Additional file 1: Tables S1, S2, S3 show results from alternative models using a continuous treated net coverage exposure variable rather than a binary comparison of 50% and more vs. less than 50% of usage. Additional file 1: Tables S4, S5, S6 show results from the always vs. never user comparison. Both these sets of models yield almost identical results in terms of direction and precision of the estimated associations.

## Discussion

In this study, 22 years of longitudinal data were used to investigate the empirical relationship between early life exposure to malaria and adolescent socio-demographic outcomes. The results presented suggest that increased use of treated nets in early childhood was associated with substantial increases in likelihood of completing secondary school, both for men and for women. On average, less than a quarter of children using nets infrequently in childhood completed secondary school; among children using treated nets regularly, this proportion was closer to one third. Despite these relatively sizeable increases in secondary school attendance, early life exposure to malaria was only weakly associated with delays in marriage or childbearing after adjustment for potential confounders including parental education, relative wealth and year of birth. At the time of the survey, when respondents were on average 20 years old, 45% of women in the low ITN use group already had children; with high ITN use, this proportion was marginally lower at 40% [aOR 0.91 (0.69,1.19)]. In comparison, only 7% of men reported having had children at this time point in the low use group, and 6% in the high use group; it is possible that some of the family planning effects may only become evident later, especially among men, where educational attainment differences are larger. More generally, the earlier entry into marriage and childbearing among women may partially explain the more marked effects on education in men, such as for completing primary education and for remaining in school.

In terms of the main mechanism underlying the observed associations between early life malaria exposure and adult educational attainment it is important to highlight that the differences here are unrelated to the survival benefits documented in the first few years of children’s lives, because only surviving children were analysed here. While the data available does not allow to directly test this hypothesis, it seems plausible that continued protection from ITNs prevented children from recurring mild and severe (but non-lethal) malaria infections, allowing them to develop better in early childhood and to subsequently perform better in school, as already suggested in some of the existing literature [10–12].

While the data used for this study is unique, there are a few limitations worth highlighting. First, the study is observational in nature, and thus cannot be interpreted causally. Even though the risk of confounding bias should be relatively minor in this study due to the controls included in the empirical model, residual confounding cannot be ruled out—it is certainly possible that there are other factors that could have increased net use in early life and positively contributed to later life outcomes. One such factor that is not controlled for is use of ITNs in later childhood, which may help children attend school and prevent infections in later life. If parents using ITNs frequently in early life also use ITNs more frequently later, this may explain some of the benefits documented here. Second, both the initial ITN use and the schooling outcomes were reported by interviewees and not independently verified. It is possible that these self-reports were not accurate in all cases, which would induce measurement error in the estimation. Third, as already mentioned above, the data did not include final educational or sociodemographic outcomes. At the time of the survey, 90% of respondents had completed their schooling, which makes larger subsequent changes relatively unlikely for educational attainment; for fertility and marriage, the study was conducted relatively early in the reproductive lives of study participants: longer-term follow-up will be needed to assess the relationship between early life exposure to malaria and completed fertility as well as marriage rates overall. Fourth, reliable data on causes of death is not available, and thus the study cannot say much about the impact of selective mortality on observed differences in sociodemographic outcomes. Last, it is not clear how generalizable the results presented here are to other geographical or contemporary settings; the young adults analysed here were born into a context of high malaria transmission: it seems plausible that effects might be different in other contexts. It is also important to bear in mind here that the sample only includes surviving children, and thus excludes some of the children most severely affected by malaria – the analysis presented here does not allow to directly assess such differences.

## Conclusion

This long-term study of over 5000 participants in rural Tanzania found that regular use of ITNs in early life was associated with increased completion of both primary school and 4 years of secondary school. In conclusion, early-life malaria control may have long-term positive effects on educational attainment, although further research is needed to fully understand the mechanisms behind these associations, as well as to explore other effects of early-life malaria control in young adults.

## Supplementary information

**Additional file 1: Figure S1**. Reported treated net use – proportion of times visitedreported to have slept under a treated net. **Table S1**. Continuous Exposure: Early life treatednet use and Socio demographic outcomes in adulthood. **Table S2**. Continuous Exposure: Early life treated net use and Socio demographic outcomes in adulthood. **Table S3**. Continuous Exposure: Early lifetreated net use and Socio demographic outcomes in adulthood. **Table S4**. Adjusted logistic regression-All: Early life treated net use and socialdemographic outcomes in adulthood. **Table S5**. Adjusted logistic regression-Females: Early life treated net use and socialdemographic outcomes in adulthood. **Table S6**. Adjusted logistic regression-Males: Early life treated net use and socialdemographic outcomes in adulthood.

## Data Availability

The data sets used and/or analysed during the current study are available from the corresponding author upon reasonable request.
