# Supplementary material for: Associations between the use of insecticide-treated nets in early childhood and educational outcomes, marriage and child-bearing in early adulthood: evidence from a 22-year prospective cohort study in Tanzania
Source: Malar J. 2023 Apr 25;22:134. doi: 10.1186/s12936-023-04560-z (PMC10127494; doi:10.1186/s12936-023-04560-z)
Supplement: Supplementary file 1 — Additional file 1: Figure S1. Reported treated net use – proportion of times visitedreported to have slept under a treated net. Table S1. Continuous Exposure: Early life treatednet use and Socio demographic outcomes in adulthood. Table S2. Continuous Exposure: Early life treated net use and Socio demographic outcomes in adulthood. Table S3. Continuous Exposure: Early lifetreated net use and Socio demographic outcomes in adulthood. Table S4. Adjusted logistic regression-All: Early life treated net use and socialdemographic outcomes in adulthood. Table S5. Adjusted logistic regression-Females: Early life treated net use and socialdemographic outcomes in adulthood. Table S6. Adjusted logistic regression-Males: Early life treated net use and socialdemographic outcomes in adulthood. [file 12936_2023_4560_MOESM1_ESM.docx]

***Supplementary Materials***

******

**Figure S1: Reported treated net use – proportion of times visited reported to have slept under a treated net*.***

| **Table S1: Continuous Exposure (All): Early life treated net use and Socio demographic outcomes in adulthood** | | | | | |
| --- | --- | --- | --- | --- | --- |
| **Variables** | **(1)** | **(2)** | **(3)** | **(4)** | **(5)** |
|  | **In school** | **7 years plus** | **11 years plus** | **Married** | **Has children** |
| Treated net use | 0.0691 | 0.0509 | 0.107 | -0.0475 | -0.0306 |
|  | (0.0288 - 0.109) | (0.0218 - 0.0801) | (0.0544 - 0.161) | (-0.0874 - -0.00747) | (-0.0632 - 0.00190) |
| Untreated net use | 0.0543 | 0.0332 | 0.132 | -0.0309 | -0.0172 |
|  | (0.0104 - 0.0982) | (0.00294 - 0.0635) | (0.0774 - 0.188) | (-0.0902 - 0.0285) | (-0.0593 - 0.0248) |
| **Wealth quintile** |  |  |  |  |  |
| Wealth quintile 2 | -0.00955 | -0.0134 | -0.00425 | -0.0238 | -0.00869 |
|  | (-0.0410 - 0.0219) | (-0.0477 - 0.0209) | (-0.0344 - 0.0259) | (-0.0626 - 0.0150) | (-0.0482 - 0.0309) |
| Wealth quintile 3 | 0.00286 | 0.0201 | 0.0345 | -0.0191 | -0.0223 |
|  | (-0.0206 - 0.0264) | (-0.00669 - 0.0469) | (0.00327 - 0.0657) | (-0.0475 - 0.00919) | (-0.0553 - 0.0107) |
| Wealth quintile 4 | 0.0405 | 0.0283 | 0.112 | -0.0274 | -0.0346 |
|  | (-0.00582 - 0.0869) | (-0.00439 - 0.0609) | (0.0706 - 0.153) | (-0.0593 - 0.00455) | (-0.0746 - 0.00549) |
| Wealth quintile 5 | 0.0956 | 0.0532 | 0.181 | -0.0807 | -0.0904 |
|  | (0.0619 - 0.129) | (0.0171 - 0.0893) | (0.136 - 0.226) | (-0.115 - -0.0469) | (-0.119 - -0.0618) |
| **Parent education** |  |  |  |  |  |
| None | -0.0170 | -0.149 | -0.186 | 0.0808 | 0.00965 |
|  | (-0.0791 - 0.0451) | (-0.211 - -0.0869) | (-0.306 - -0.0650) | (0.00726 - 0.154) | (-0.0719 - 0.0912) |
| Some primary | -0.0272 | -0.0759 | -0.167 | 0.0701 | 0.00188 |
|  | (-0.0985 - 0.0440) | (-0.122 - -0.0294) | (-0.299 - -0.0340) | (0.00988 - 0.130) | (-0.0872 - 0.0909) |
| Primary completed | 0.0414 | -0.0227 | -0.0304 | 0.00475 | -0.0597 |
|  | (-0.0168 - 0.0997) | (-0.0755 - 0.0301) | (-0.141 - 0.0796) | (-0.0497 - 0.0592) | (-0.131 - 0.0118) |
| Secondary plus | 0.211 | 0.00688 | 0.149 | -0.0598 | -0.0912 |
|  | (0.123 - 0.299) | (-0.0665 - 0.0802) | (-0.0166 - 0.314) | (-0.144 - 0.0246) | (-0.186 - 0.00341) |
| **Year of birth** |  |  |  |  |  |
| Cohort 1999 | 0.0366 | -0.00915 | -0.0164 | -0.0391 | -0.0794 |
|  | (0.0134 - 0.0599) | (-0.0290 - 0.0107) | (-0.0433 - 0.0106) | (-0.0647 - -0.0135) | (-0.102 - -0.0566) |
| Cohort 2000 | 0.0952 | -0.0278 | -0.0449 | -0.106 | -0.144 |
|  | (0.0634 - 0.127) | (-0.0569 - 0.00122) | (-0.0771 - -0.0128) | (-0.126 - -0.0855) | (-0.169 - -0.119) |
| Observations | 5,073 | 5,073 | 5,073 | 5,073 | 5,073 |
| R-squared | 0.066 | 0.057 | 0.109 | 0.151 | 0.207 |

*Liner regression model adjusted for treated net use, wealth quintile, caregiver education and year of birth. Column (1), (2), (3), (4) and (5) display the coefficients of adjusted linear regression with 95% confidence intervals in parentheses

| **Table S2: Continuous Exposure (Females): Early life treated net use and Socio demographic outcomes in adulthood** | | | | | |
| --- | --- | --- | --- | --- | --- |
| **Variables** | (1) | (2) | (3) | (4) | (5) |
|  | In school | 7 years plus | 11 years plus | Married | Has children |
| Treated net use | 0.0460 | 0.00915 | 0.0838 | -0.0641 | -0.0573 |
|  | (-0.0123 - 0.104) | (-0.0242 - 0.0425) | (0.0249 - 0.143) | (-0.154 - 0.0264) | (-0.116 - 0.00122) |
| Untreated net use | 0.0651 | 0.00264 | 0.118 | -0.0246 | -0.0183 |
|  | (0.00252 - 0.128) | (-0.0397 - 0.0450) | (0.0565 - 0.180) | (-0.148 - 0.0987) | (-0.0986 - 0.0621) |
| **Wealth quintile** |  |  |  |  |  |
| Wealth quintile 2 | 0.0130 | -0.00717 | -0.0124 | -0.0265 | -0.0145 |
|  | (-0.0227 - 0.0487) | (-0.0436 - 0.0292) | (-0.0634 - 0.0386) | (-0.0843 - 0.0313) | (-0.0883 - 0.0592) |
| Wealth quintile 3 | -0.000682 | 0.00411 | 0.0366 | -0.0114 | -0.0294 |
|  | (-0.0353 - 0.0340) | (-0.0245 - 0.0328) | (0.00704 - 0.0662) | (-0.0541 - 0.0313) | (-0.0871 - 0.0284) |
| Wealth quintile 4 | 0.0187 | -0.00264 | 0.113 | -0.0461 | -0.0541 |
|  | (-0.0347 - 0.0721) | (-0.0362 - 0.0309) | (0.0444 - 0.182) | (-0.0952 - 0.00313) | (-0.136 - 0.0281) |
| Wealth quintile 5 | 0.0860 | 0.0209 | 0.175 | -0.128 | -0.159 |
|  | (0.0425 - 0.130) | (-0.0134 - 0.0553) | (0.128 - 0.222) | (-0.181 - -0.0748) | (-0.205 - -0.112) |
| **Parent education** |  |  |  |  |  |
| None | -0.000631 | -0.0923 | -0.133 | 0.102 | 0.0592 |
|  | (-0.0948 - 0.0935) | (-0.194 - 0.00982) | (-0.339 - 0.0729) | (-0.0320 - 0.236) | (-0.0975 - 0.216) |
| Some primary | -0.0166 | -0.0451 | -0.102 | 0.0567 | 0.0136 |
|  | (-0.0967 - 0.0635) | (-0.125 - 0.0344) | (-0.313 - 0.108) | (-0.0724 - 0.186) | (-0.133 - 0.160) |
| Primary completed | 0.0720 | 0.0112 | 0.0480 | -0.0233 | -0.0759 |
|  | (-0.00414 - 0.148) | (-0.0628 - 0.0852) | (-0.147 - 0.243) | (-0.142 - 0.0953) | (-0.211 - 0.0590) |
| Secondary plus | 0.202 | 0.0392 | 0.162 | -0.122 | -0.124 |
|  | (0.0701 - 0.335) | (-0.0525 - 0.131) | (-0.0242 - 0.348) | (-0.273 - 0.0288) | (-0.284 - 0.0352) |
| **Year of birth** |  |  |  |  |  |
| Cohort 1999 | 0.0323 | -0.0110 | 0.000659 | -0.0607 | -0.120 |
|  | (0.00311 - 0.0615) | (-0.0297 - 0.00779) | (-0.0455 - 0.0468) | (-0.100 - -0.0210) | (-0.169 - -0.0712) |
| Cohort 2000 | 0.0966 | -0.0205 | -0.0345 | -0.136 | -0.216 |
|  | (0.0552 - 0.138) | (-0.0518 - 0.0107) | (-0.0753 - 0.00638) | (-0.180 - -0.0924) | (-0.265 - -0.168) |
| Observations | 2,567 | 2,567 | 2,567 | 2,567 | 2,567 |
| R-squared | 0.064 | 0.039 | 0.104 | 0.065 | 0.076 |

*Liner regression model adjusted for treated net use, wealth quintile, caregiver education and year of birth. Column (1), (2), (3), (4) and (5) display the coefficients of adjusted linear regression with 95% confidence intervals in parentheses

| **Table S3: Continuous Exposure (Males): Early life treated net use and Socio demographic outcomes in adulthood** | | | | | |
| --- | --- | --- | --- | --- | --- |
| **Variables** | **(1)** | **(2)** | **(3)** | **(4)** | **(5)** |
|  | **In school** | **7 years plus** | **11 years plus** | **Married** | **Has children** |
| Treated net use | 0.0906 | 0.0903 | 0.128 | -0.0345 | -0.00962 |
|  | (0.0486 - 0.133) | (0.0417 - 0.139) | (0.0575 - 0.199) | (-0.0841 - 0.0152) | (-0.0498 - 0.0305) |
| Untreated net use | 0.0411 | 0.0603 | 0.145 | -0.0505 | -0.0270 |
|  | (-0.0332 - 0.115) | (0.0243 - 0.0962) | (0.0693 - 0.220) | (-0.113 - 0.0115) | (-0.0599 - 0.00599) |
| **Wealth quintile** |  |  |  |  |  |
| Wealth quintile 2 | -0.0309 | -0.0177 | 0.00391 | -0.0213 | -0.000280 |
|  | (-0.0860 - 0.0241) | (-0.0719 - 0.0364) | (-0.0304 - 0.0382) | (-0.0665 - 0.0239) | (-0.0435 - 0.0429) |
| Wealth quintile 3 | 0.0105 | 0.0393 | 0.0380 | -0.0289 | -0.0137 |
|  | (-0.0393 - 0.0604) | (-0.000457 - 0.0790) | (-0.0103 - 0.0863) | (-0.0618 - 0.00414) | (-0.0440 - 0.0165) |
| Wealth quintile 4 | 0.0611 | 0.0587 | 0.113 | -0.0117 | -0.0207 |
|  | (0.0106 - 0.112) | (0.0107 - 0.107) | (0.0622 - 0.163) | (-0.0439 - 0.0205) | (-0.0410 - -0.000398) |
| Wealth quintile 5 | 0.106 | 0.0853 | 0.183 | -0.0309 | -0.0180 |
|  | (0.0561 - 0.156) | (0.0427 - 0.128) | (0.117 - 0.249) | (-0.0743 - 0.0125) | (-0.0510 - 0.0150) |
| **Parent education** |  |  |  |  |  |
| None | -0.0144 | -0.196 | -0.223 | 0.0481 | -0.0473 |
|  | (-0.103 - 0.0743) | (-0.274 - -0.119) | (-0.392 - -0.0536) | (0.00911 - 0.0870) | (-0.135 - 0.0404) |
| Some primary | -0.0179 | -0.0953 | -0.215 | 0.0803 | -0.00877 |
|  | (-0.123 - 0.0871) | (-0.140 - -0.0501) | (-0.372 - -0.0584) | (0.0242 - 0.136) | (-0.102 - 0.0841) |
| Primary completed | 0.0331 | -0.0434 | -0.0920 | 0.0310 | -0.0394 |
|  | (-0.0586 - 0.125) | (-0.0974 - 0.0107) | (-0.230 - 0.0465) | (-0.0166 - 0.0785) | (-0.116 - 0.0370) |
| Secondary plus | 0.249 | -0.0195 | 0.151 | 0.00442 | -0.0376 |
|  | (0.127 - 0.371) | (-0.120 - 0.0809) | (-0.0866 - 0.389) | (-0.0523 - 0.0611) | (-0.145 - 0.0696) |
| **Year of birth** |  |  |  |  |  |
| Cohort 1999 | 0.0387 | -0.00638 | -0.0384 | -0.0135 | -0.0351 |
|  | (-0.000824 - 0.0783) | (-0.0456 - 0.0328) | (-0.0748 - -0.00199) | (-0.0489 - 0.0219) | (-0.0612 - -0.00902) |
| Cohort 2000 | 0.0918 | -0.0366 | -0.0574 | -0.0719 | -0.0678 |
|  | (0.0535 - 0.130) | (-0.0883 - 0.0151) | (-0.105 - -0.00942) | (-0.0991 - -0.0447) | (-0.0849 - -0.0507) |
| Observations | 2,506 | 2,506 | 2,506 | 2,506 | 2,506 |
| R-squared | 0.078 | 0.070 | 0.126 | 0.036 | 0.027 |

*Liner regression model adjusted for treated net use, wealth quintile, caregiver education and year of birth. Column (1), (2), (3), (4) and (5) display the coefficients of adjusted linear regression with 95% confidence intervals in parentheses

| **Table S4: Adjusted logistic regression-All: Early life treated net use and social demographic outcomes in adulthood** | | | | | |
| --- | --- | --- | --- | --- | --- |
| **Variables** | **(1)** | **(2)** | **(3)** | **(4)** | **(5)** |
|  | **In school** | **7 years plus** | **11 years plus** | **Married** | **Has children** |
| Treated net use | 1.741 | 1.528 | 1.837 | 0.687 | 0.881 |
|  | (1.125 - 2.697) | (1.037 - 2.252) | (1.366 - 2.470) | (0.469 - 1.005) | (0.608 - 1.278) |
| Untreated net use | 1.350 | 1.313 | 1.773 | 0.834 | 1.022 |
|  | (0.853 - 2.136) | (0.916 - 1.883) | (1.313 - 2.395) | (0.546 - 1.275) | (0.666 - 1.569) |
| **Wealth quintile** |  |  |  |  |  |
| Wealth quintile 2 | 0.863 | 0.958 | 0.998 | 0.877 | 0.998 |
|  | (0.593 - 1.255) | (0.595 - 1.541) | (0.681 - 1.463) | (0.675 - 1.140) | (0.750 - 1.329) |
| Wealth quintile 3 | 1.065 | 1.646 | 1.560 | 0.992 | 0.745 |
|  | (0.795 - 1.427) | (1.191 - 2.275) | (1.150 - 2.116) | (0.780 - 1.262) | (0.573 - 0.968) |
| Wealth quintile 4 | 1.025 | 1.513 | 2.000 | 0.766 | 0.737 |
|  | (0.601 - 1.751) | (0.935 - 2.447) | (1.367 - 2.926) | (0.569 - 1.032) | (0.550 - 0.989) |
| Wealth quintile 5 | 1.587 | 1.893 | 2.329 | 0.697 | 0.557 |
|  | (1.094 - 2.304) | (1.190 - 3.010) | (1.562 - 3.472) | (0.541 - 0.897) | (0.426 - 0.727) |
| **Parent education** |  |  |  |  |  |
| None | 0.626 | 0.141 | 0.260 | 2.410 | 1.240 |
|  | (0.343 - 1.142) | (0.0394 - 0.505) | (0.120 - 0.563) | (1.419 - 4.093) | (0.741 - 2.074) |
| Some primary | 0.424 | 0.250 | 0.433 | 2.153 | 1.201 |
|  | (0.212 - 0.849) | (0.0748 - 0.834) | (0.202 - 0.931) | (1.319 - 3.514) | (0.737 - 1.957) |
| Primary completed | 1.083 | 0.463 | 0.952 | 1.209 | 0.765 |
|  | (0.592 - 1.984) | (0.125 - 1.715) | (0.590 - 1.536) | (0.754 - 1.937) | (0.494 - 1.185) |
| Secondary plus | 2.726 | 1.017 | 1.845 | 0.345 | 0.514 |
|  | (1.289 - 5.761) | (0.120 - 8.580) | (0.976 - 3.486) | (0.142 - 0.838) | (0.224 - 1.179) |
| **Year of birth** |  |  |  |  |  |
| Cohort 1999 | 1.289 | 1.227 | 0.949 | 0.859 | 0.643 |
|  | (0.989 - 1.678) | (0.871 - 1.729) | (0.745 - 1.209) | (0.643 - 1.148) | (0.513 - 0.806) |
| Cohort 2000 | 1.863 | 0.887 | 0.753 | 0.529 | 0.356 |
|  | (1.423 - 2.438) | (0.588 - 1.339) | (0.634 - 0.895) | (0.373 - 0.751) | (0.275 - 0.462) |
| **Observations** | 2,141 | 2,152 | 2,170 | 2,170 | 2,170 |

* Column (1), (2), (3), (4) and (5) represents multiple logistic regress models for always using treated nets versus never using treated nets for both females and males. Each model has been adjusted for treated net use, wealth quintile, caregiver education and year of birth with odds ratio and 95% confidence intervals in parentheses

| **Table S5: Adjusted logistic regression-Females: Early life treated net use and social demographic outcomes in adulthood** | | | | | |
| --- | --- | --- | --- | --- | --- |
| **Variables** | **(1)** | **(2)** | **(3)** | **(4)** | **(5)** |
|  | **In school** | **7 years plus** | **11 years plus** | **Married** | **Has children** |
| Treated net use | 1.480 | 0.837 | 1.635 | 0.696 | 0.927 |
|  | (0.822 - 2.665) | (0.485 - 1.446) | (1.103 - 2.424) | (0.410 - 1.184) | (0.566 - 1.519) |
| Untreated net use | 1.582 | 0.862 | 1.559 | 0.957 | 1.248 |
|  | (0.862 - 2.901) | (0.471 - 1.579) | (1.100 - 2.209) | (0.539 - 1.700) | (0.716 - 2.177) |
| **Wealth quintile** |  |  |  |  |  |
| Wealth quintile 2 | 1.105 | 0.913 | 0.933 | 0.837 | 0.950 |
|  | (0.555 - 2.200) | (0.472 - 1.768) | (0.520 - 1.677) | (0.632 - 1.108) | (0.715 - 1.261) |
| Wealth quintile 3 | 1.102 | 1.354 | 1.564 | 1.003 | 0.676 |
|  | (0.597 - 2.034) | (0.866 - 2.115) | (1.120 - 2.185) | (0.754 - 1.334) | (0.476 - 0.960) |
| Wealth quintile 4 | 1.056 | 1.271 | 1.846 | 0.733 | 0.703 |
|  | (0.426 - 2.621) | (0.677 - 2.388) | (1.077 - 3.165) | (0.505 - 1.062) | (0.533 - 0.928) |
| Wealth quintile 5 | 1.853 | 1.443 | 2.059 | 0.661 | 0.492 |
|  | (0.992 - 3.463) | (0.747 - 2.786) | (1.408 - 3.011) | (0.415 - 1.052) | (0.334 - 0.725) |
| **Parent education** |  |  |  |  |  |
| None | 0.518 | 0.217 | 0.520 | 2.116 | 1.207 |
|  | (0.162 - 1.662) | (0.0460 - 1.027) | (0.162 - 1.672) | (1.132 - 3.957) | (0.671 - 2.171) |
| Some primary | 0.548 | 0.264 | 0.815 | 1.620 | 0.989 |
|  | (0.196 - 1.528) | (0.0605 - 1.150) | (0.230 - 2.885) | (0.842 - 3.118) | (0.566 - 1.728) |
| Primary completed | 1.504 | 0.842 | 1.886 | 1.033 | 0.648 |
|  | (0.549 - 4.122) | (0.205 - 3.464) | (0.683 - 5.207) | (0.573 - 1.863) | (0.384 - 1.093) |
| Secondary plus | 3.087 |  | 2.766 | 0.330 | 0.453 |
|  | (0.881 - 10.81) |  | (0.988 - 7.748) | (0.130 - 0.838) | (0.167 - 1.234) |
| **Year of birth** |  |  |  |  |  |
| Cohort 1999 | 1.125 | 1.476 | 0.964 | 0.924 | 0.659 |
|  | (0.715 - 1.769) | (0.915 - 2.379) | (0.685 - 1.357) | (0.652 - 1.310) | (0.509 - 0.855) |
| Cohort 2000 | 2.060 | 1.213 | 0.752 | 0.644 | 0.362 |
|  | (1.331 - 3.189) | (0.656 - 2.243) | (0.511 - 1.106) | (0.444 - 0.935) | (0.283 - 0.463) |
| **Observations** | 1,046 | 986 | 1,071 | 1,071 | 1,066 |

* Column (1), (2), (3), (4) and (5) represents multiple logistic regress models for always using treated nets versus never using treated nets for females. Each model has been adjusted for treated net use, wealth quintile, caregiver education and year of birth with odds ratio and 95% confidence intervals in parentheses

| **Table S6: Adjusted logistic regression-Males: Early life treated net use and social demographic outcomes in adulthood** | | | | | |
| --- | --- | --- | --- | --- | --- |
| **Variables** | **(1)** | **(2)** | **(3)** | **(4)** | **(5)** |
|  | **In school** | **7 years plus** | **11 years plus** | **Married** | **Has children** |
| Treated net use | 2.124 | 2.160 | 2.153 | 0.782 | 0.822 |
|  | (1.342 - 3.360) | (1.211 - 3.850) | (1.475 - 3.141) | (0.325 - 1.884) | (0.422 - 1.599) |
| Untreated net use | 1.304 | 1.556 | 2.166 | 0.524 | 0.493 |
|  | (0.626 - 2.718) | (1.031 - 2.347) | (1.382 - 3.396) | (0.118 - 2.322) | (0.227 - 1.069) |
| **Wealth quintile** |  |  |  |  |  |
| Wealth quintile 2 | 0.644 | 1.053 | 1.017 | 1.069 | 1.313 |
|  | (0.384 - 1.080) | (0.540 - 2.052) | (0.621 - 1.668) | (0.593 - 1.929) | (0.498 - 3.463) |
| Wealth quintile 3 | 0.998 | 1.920 | 1.608 | 0.833 | 0.965 |
|  | (0.591 - 1.688) | (1.090 - 3.382) | (0.994 - 2.602) | (0.377 - 1.839) | (0.431 - 2.165) |
| Wealth quintile 4 | 0.969 | 1.865 | 2.137 | 1.268 | 1.006 |
|  | (0.517 - 1.817) | (0.838 - 4.150) | (1.114 - 4.100) | (0.475 - 3.389) | (0.429 - 2.356) |
| Wealth quintile 5 | 1.393 | 2.358 | 2.612 | 0.798 | 0.879 |
|  | (0.752 - 2.579) | (1.312 - 4.239) | (1.424 - 4.794) | (0.280 - 2.275) | (0.455 - 1.697) |
| **Parent education** |  |  |  |  |  |
| None | 0.776 | 0.0748 | 0.163 | 5.922 | 1.424 |
|  | (0.327 - 1.842) | (0.00959 - 0.583) | (0.0501 - 0.532) | (1.132 - 30.97) | (0.215 - 9.435) |
| Some primary | 0.386 | 0.177 | 0.293 | 10.72 | 3.524 |
|  | (0.149 - 0.999) | (0.0217 - 1.447) | (0.119 - 0.720) | (2.179 - 52.75) | (0.950 - 13.06) |
| Primary completed | 0.896 | 0.249 | 0.590 | 3.427 | 1.905 |
|  | (0.392 - 2.045) | (0.0308 - 2.008) | (0.322 - 1.079) | (0.648 - 18.13) | (0.446 - 8.135) |
| Secondary plus | 2.689 | 0.353 | 1.924 |  | 1.331 |
|  | (0.803 - 9.011) | (0.0233 - 5.347) | (0.570 - 6.492) |  | (0.123 - 14.41) |
| **Year of birth** |  |  |  |  |  |
| Cohort 1999 | 1.472 | 1.073 | 0.930 | 0.705 | 0.526 |
|  | (0.895 - 2.420) | (0.587 - 1.963) | (0.675 - 1.281) | (0.306 - 1.625) | (0.244 - 1.134) |
| Cohort 2000 | 1.799 | 0.695 | 0.752 | 0.178 | 0.326 |
|  | (1.231 - 2.629) | (0.361 - 1.336) | (0.517 - 1.093) | (0.0767 - 0.412) | (0.165 - 0.644) |
| **Observations** | 1,061 | 1,091 | 1,095 | 999 | 1,018 |

* Column (1), (2), (3), (4) and (5) represents multiple logistic regress models for always using treated nets versus never using treated nets for males. Each model has been adjusted for treated net use, wealth quintile, caregiver education and year of birth with odds ratio and 95% confidence intervals in parentheses
